# Injectable wireless microdevices: challenges and opportunities

**DOI:** 10.1186/s42234-021-00080-w

**Published:** 2021-12-23

**Authors:** Adam Khalifa, Sunwoo Lee, Alyosha Christopher Molnar, Sydney Cash

**Affiliations:** 1 Department of Neurology, Massachusetts General Hospital, Harvard Medical School, Boston, MA USA; 2 Department of Electrical and Computer Engineering, Cornell University, Ithaca, NY USA

**Keywords:** Microscale, Wireless, Injectable, Minimally-invasive, Neural interfaces, Autonomous microsystems

## Abstract

In the past three decades, we have witnessed unprecedented progress in wireless implantable medical devices that can monitor physiological parameters and interface with the nervous system. These devices are beginning to transform healthcare. To provide an even more stable, safe, effective, and distributed interface, a new class of implantable devices is being developed; injectable wireless microdevices. Thanks to recent advances in micro/nanofabrication techniques and powering/communication methodologies, some wireless implantable devices are now on the scale of dust (< 0.5 mm), enabling their full injection with minimal insertion damage. Here we review state-of-the-art fully injectable microdevices, discuss their injection techniques, and address the current challenges and opportunities for future developments.

## Introduction

Implantable medical devices (IMDs) encompass a wide range of applications such as neural network modulation (Barbruni et al. 2020), brain activity recording (Liu et al. 2020), drug delivery (Khan et al. 2019), temperature monitoring (Shi et al. 2021), and glucose sensing (Mujeeb-U-Rahman et al. 2019). The demand for battery-free wirelessly powered IMDs is rapidly growing (Yang et al. 2020; Datta-Chaudhuri 2021) as they have reduced encapsulation requirements and they eliminate the need for percutaneous wires that can cause infections and other complications in patients. Furthermore, wireless implants allow researchers to study freely-behaving animals that can move and interact socially in more natural environments (Y. Yang et al. 2021).

**Fig. 1:**
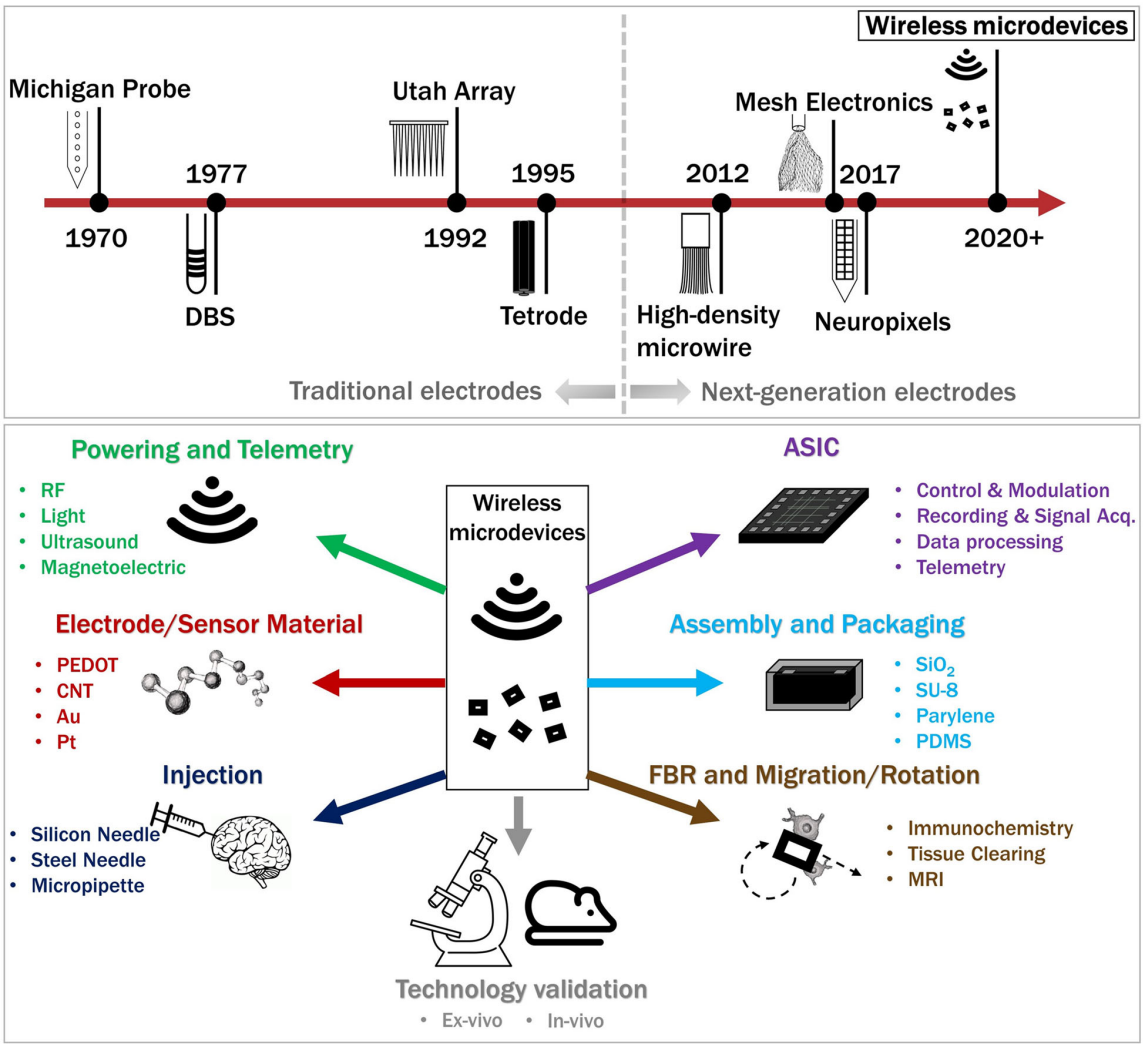
An overview of injectable wireless microdevices. (Top) An evolutionary timeline of intracortical and depth electrodes that includes wireless microdevices customized for neural interfaces. (Bottom) The interdisciplinary research enabling injectable wireless microdevices

Recent advances in complementary metal-oxide-semiconductor (CMOS) technology for highly miniaturized and low-power integrated circuits, novel micro/nanofabrication techniques, and superior wireless links together provide an opportunity to eliminate batteries and to create implantable microdevices that integrate all the components needed to interface with the nervous system and monitor a wide variety of physiological parameters. These wireless free-floating implantable devices have been referred to as wireless motes, dust, and microdevices. In the field of neural interfaces, they represent the latest generation of electrodes as shown in Fig. 1 (Top).

The advantages of stand-alone injectable microdevices over conventional IMDs include:

1. Ability to choose from a wide range of configurations and site locations. For instance, microdevices can be distributed injected across multiple regions in the brain and reach areas inaccessible to conventional IMDs (e.g., folded cortices), providing clinicians and researchers higher specificity in their treatments or experiments.
2. Microdevices allow minimally-invasive implantation techniques (e.g., laparoscopy or stereotaxic injection) which is expected to significantly reduce the immune or foreign body response (FBR) as they avoid the risks of surgical complications associated with open craniotomy and extensive dissection (Polyzos et al. 2015; Darouiche 2004). An example of a complication is a bacterial infection arising when the meningeal layer is exposed during craniotomy (Kourbeti et al. 2015).
3. Injectable microdevices are well suited for chronic applications that require long-term stability as they are not expected to suffer from micromotion, which is known to cause inflammation and scar formation (astrogliosis) around the implant (Biran et al. 2007; Ersen et al. 2015).

In summary, injectable microdevices de-risk consequences of chronic as well as acute damages associated with surgeries, paving a path for new treatments, and expanding the targetable patient population.

In this review, we define injectable wireless microdevices as follows:

1. They must be microscale (< 0.2 mm^3^) and injectable using appropriate injection tools.
2. The implants must be powered wirelessly. Therefore, technologies such as the syringe injectable mesh electronics are excluded in this review.
3. The microdevices must contain active circuitry. Thus, injectable nanoelectrodes or nanoparticles are excluded.

In general, an injectable microdevice includes at least (Fig. 1 (Bottom)) a receiver to wirelessly capture power, a pair of electrodes or a sensor, an electronic circuit (often integrated circuit), and encapsulation to shield the electronics from biological media.

## Fully injectable microdevices and injection strategies

Here we summarize five injectable microdevices that have been developed in the last 3 years and have been validated in animal models. We also describe associated injection methodologies that are becoming increasingly critical. To the best of our knowledge, no other injectable implants satisfy the criteria stated above.

### Optical wireless integrated circuit (OWIC)

**Fig. 2:**
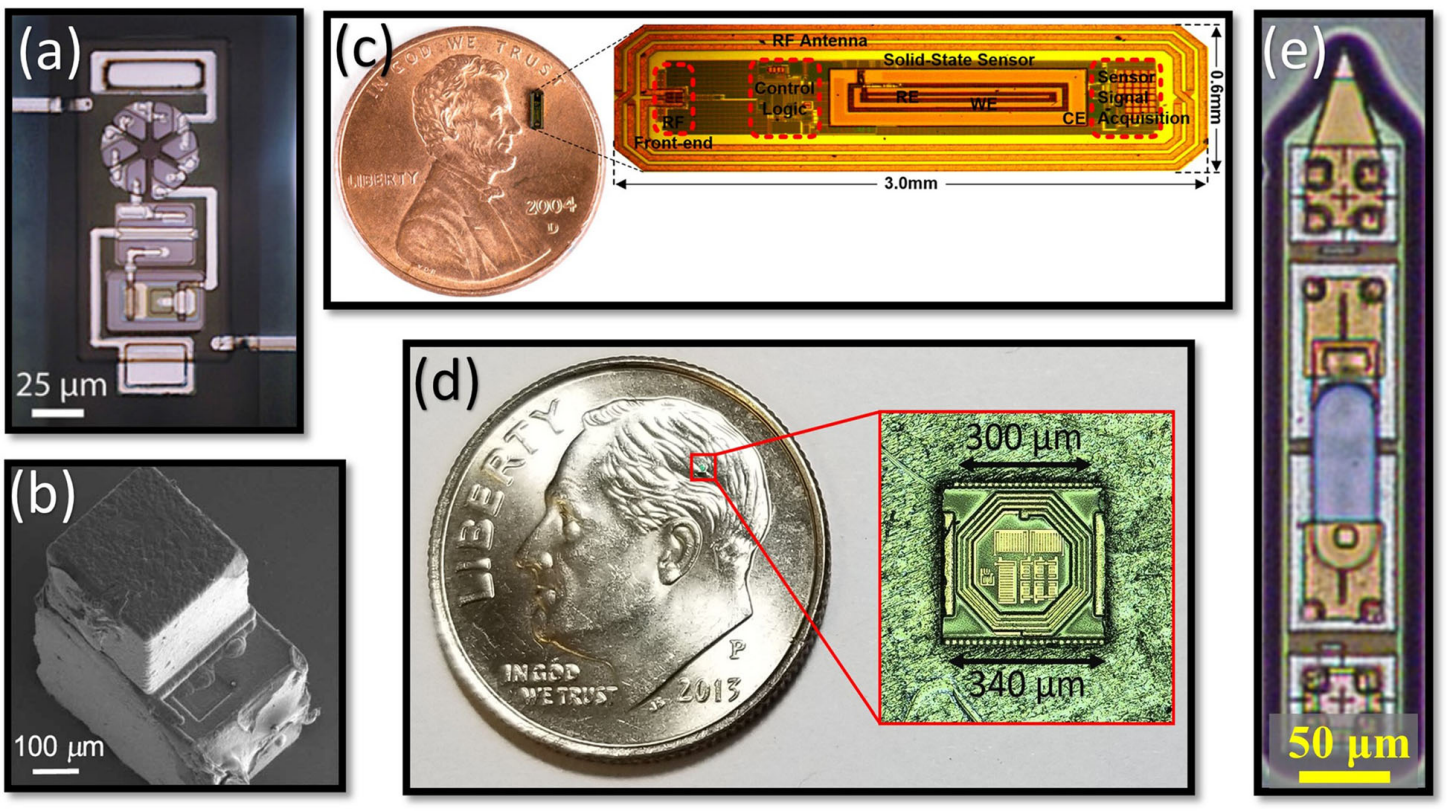
State-of-the-art fully injectable wireless microdevices: **a** an OWIC (Cortese et al. 2020), **b** a wireless temperature sensor (Shi et al. 2021), **c** a wireless glucose sensor (Mujeeb-U-Rahman et al. 2019), **d** a Microbead (reprinted with permission from [X], Copyright 2019, IEEE), and **e** a MOTE (S. Lee et al. 2020)

Cortese et al. have demonstrated a microdevice for temperature sensing (Fig. 2(a)) (Cortese et al. 2020). The optical wireless integrated circuit (OWIC consists of an AlGaAs micro-scale light-emitting diode (μ-LED) heterogeneously integrated with silicon (Si) diodes. In the OWIC temperature sensor, four serially connected Si diodes act as photovoltaic (PV) to power the AlGaAs μLED. Because the Si PV voltage depends on temperature, the AlGaAs μLED emission intensity tracks the temperature change. Atomic layer deposition (ALD) SiO_2_, plasma-enhanced chemical vapor deposition (PECVD) Si_X_N_Y_, and SU-8 (together a few μm thick) were used for encapsulation. While this simple construction makes the integration fabrication easier, such amplitude modulation leads to a high noise floor and is also prone to environmental fluctuations. Its injector tool is a micromachined silicon microneedle that securely holds the implant in a recess with polyethylene glycol (PEG) as a temporary adhesive. Once inside the brain, the PEG is dissolved by intercellular fluid, and the OWIC is mechanically pushed out by a secondary needle (Cortese et al. 2020). While being the smallest microdevice, the OWIC’s insertion technique is limited by its custom-made needles that cannot be shared between different microdevices. Furthermore, the insertion technique does not allow the microdevice to be oriented parallel to the brain surface.

### Relaxation oscillator based temperature sensor

Shi, et al. have also demonstrated a microdevice for temperature sensing (Fig. 2(b)) (Shi et al. 2021). This 0.1 mm^3^ microdevice is manufactured in a 180 nm bulk CMOS process and is integrated with a microscale piezoelectric transducer allowing it to be powered using an ultrasound imaging probe. The subthreshold operation of the IC reduces the power consumption down to ~ 0.8 nW. Its circuitry includes a relaxation oscillator that exploits the temperature dependence of the leakage current in transistors. An 8 μm thick parylene-C coating is deposited over the microdevice for encapsulation. The sensors were deployed in a mouse brain and hindlimb to measure core body temperatures. The authors have shown that the devices are small enough to be loaded into a 1-ml syringe (with an 18G needle) filled with PBS for injection delivery.

### Glucose sensor

Munjeeb-U-Rahman et al. have reported a miniature CMOS-based glucose sensor (Fig. 2(c)) for artificial pancreas applications (Mujeeb-U-Rahman et al. 2019). The subdermal implant provides real-time glucose levels to an insulin infusion system. The device uses a solid-state electrochemical sensor created by lithographic postprocessing. A 2 cm diameter transmitter coil powers the implant at 900 MHz, with an 0.1% efficiency at 1.5 mm implantation depth. The system was verified in vivo using rat and swine models. An applicator based on an 18G needle inserts the implant in the subcutaneous space with minimal tissue damage. Once the needle is inside the tissue, the implant is pushed out using a trocar. A thin biocompatible thread is attached to the implant for extraction.

### Microbead

The Microbead is an example of a stimulating microdevice (Khalifa et al. 2019). The microdevice is implemented in a 130 nm CMOS technology with the following characteristics: 300 μm × 300 μm × 80 μm in size; a highly optimized two-coil inductive link; and integrated circuit, electrodes, and coil (Fig. 2(d)). The microbead has been extensively miniaturized by simplifying the system architecture, using aggressive layout design techniques, and implementing a novel electrode integration technique. The microbead was fully implanted in the sciatic nerve of a rat to confirm its ability to elicit action potentials in axons. Its injection tool and approach are described in detail in (Khalifa et al. 2021a). The main components of the injection tool are a 23G needle and a stainless-steel cylindrical rod, where PEG is used to temporarily affix the microdevice to the tip of the rod. The simplicity of this approach allows the insertion tool to be used for any free-floating implantable device, regardless of shape and volume, and enables the microdevice to be injected at any location and depth into the central nervous system (CNS) or peripheral nervous system (PNS). To quantitatively evaluate the needle injection tool and the delivery approach, the authors have examined the spatial precision and rotational alignment of the microdevices injected into agarose and rat brains with the aid of tissue clearing techniques and MRI.

### Microscale opto-electrically transduced electrode (MOTE)

The MOTE is a neural recording microdevice that has an AlGaAs μLED integrated on 180 nm CMOS (Fig. 2(e)) (S. Lee et al. 2020). By utilizing light as power and communication media, the MOTE achieves an impressive scaling: 330 μm × 80 μm × 30 μm, less than a single nanoliter. An external red (623 nm) LED powers the MOTE and CMOS circuits then amplify and encode measured neural signals in pulse position modulation (PPM). The PPM pulses are then emitted through the AlGaAs μLED at a longer wavelength (825 nm) and detected by an external photodetector. The MOTEs also utilize ALD and PECVD SiO_2_, Si_X_N_Y_, and Al_2_O_3_ (total thickness ~ 1 μm) for encapsulation as in OWIC and achieve several months of lifetime in the mouse brain. For manipulation, the authors use pulled micropipettes (μ-pipettes) in conjunction with a nanoinjector. A MOTE, once dispersed in a solution (saline or isopropanol) post-fabrication, is pulled in by a μ-pipette. After the solution in the μ-pipette dries, the MOTE is pushed out by the nanoinjector needle. Because the MOTEs are made to be sharp-edged, MOTEs can penetrate the dura. In other words, the incision damage is not limited by the insertion tool (e.g., μ-pipette) dimension, but by the size of the MOTE itself.

Table 1 shows that almost all microdevices are based on a CMOS application-specific integrated circuit (ASIC) which offers extreme scaling and very low power consumption. Yet, a notable disadvantage in ASICs is their high microfabrication costs. Unfortunately, there are no viable alternatives to CMOS as other technologies (e.g., off-the-shelf ICs and surface mount components on PCB) lead to bulkier implants. Nonetheless, the unit cost of ASICs will drop precipitously once the production volume increases. Table 1 also indicates that, although multiple wireless powering modalities exist in the sub-mm scale, near/mid-field RF, ultrasonic, and optical are the most promising (Khalif et al. 2021; Singer and Robinson 2021; Cai and Gutruf 2021; Won et al. 2021). Each method offers trade-offs, and thus the best powering mechanism will depend on the application. The optical powering offers excellent size scalability, while the ultrasonic powering offers the best power transfer efficiency. Near/Mid-field powering seems to provide a good balance between the two.

**Table 1:** Comparison table of the recent wireless and fully injectable microdevices that have been validated in animal models

References	Cortese et al. 2020	Shi et al. 2021	Mujeeb-U-Rahman et al. 2019	Khalifa et al. 2019	Lee et al. 2020
Device Name	OWIC	N/A	N/A	Microbead	MOTE
Application	Temperature	Temperature	Glucose	Stimulation	Recording
Wireless Link	Light	US	RF	RF	Light
CMOS Process	N/A	TSMC	TSMC	IBM 130 nm	TSMC 180 nm
Power consumption (μW)	10	< 1	< 5	< 50	< 1
Encapsulation Material	SiO_2_ and SU-8	Parylene-C	Polyurethane	SiO_2_ and SU-8	SiO_2_, Si_3_N_4_, and Al_2_O_3_
Injection Method	Micromachine silicon needle and PEG	1-ml syringe with an 18G needle	Trocar, syringe with a 18G needle	Steel rod, 23G needle, and PEG	Pulled micropipettes, nanoinjectors
Animal Model	Mouse brain	Mouse brain	Rat, Swine	Rat sciatic	Mouse brain
Volume (mm^3^)	0.0001	0.1	0.196	0.009	0.0008

While there are several other examples of wireless implants that have been aggressively miniaturized (Piech et al. 2020; Ghanbari et al. 2019; Lee et al. 2021b; Charthad et al. 2018; Ahmadi et al. 2019; Freeman et al., 2017, b; Lim et al. 2021; Yeon et al. 2016; Biederman et al. 2015; Lee et al., 2021a; Sonmezoglu et al. 2021; Freeman et al., 2017, b; Seymour et al. 2014), these devices have much lower integration levels than those described above and thus would displace much larger volumes of tissue if they were to be injected. In addition, many of them require a relay system to increase implantation depth. Unfortunately, such relay systems are often bulky and must be placed on the cortical or pial surface, nullifying the core benefit of injectable implants (i.e., minimal surgical damage).

## Challenges and Progress

The examples described above demonstrate tremendous progress in developing injectable microdevices. Continued miniaturization and optimization of such microdevices would allow: i) more floating microdevices to be concurrently inserted into the nervous system, ii) higher precision in targeting specific brain regions or nerve fiber bundles, iii) better compatibility with minimally invasive implantation procedures, leading to faster recovery time and lower risks, and iv) a reduction in FBR. Unfortunately, the development of these ultra-small devices is a laborious interdisciplinary endeavor that requires an intimate interplay between multiple engineering and science disciplines. In this section, we outline some of the challenges and progress towards developing future wireless microdevices.

### Wireless link

The major technological bottleneck in miniaturizing microdevices is the inevitable decrease in power transfer efficiency (PTE), which limits the implantation depth and/or requires large transmitted power. Furthermore, as the microdevice volume continues to shrink, the energy harvested will decrease, leading to the reduction in the supply voltage. This challenge is more pronounced in stimulating implants that require a certain voltage across their electrode pair. With the limited area, stimulating electrodes will have to utilize novel rough materials with large charge-injection capacity and small impedance (Pranti et al. 2018; Khalifa et al. 2015; Zheng et al. 2021).

Aligning the microdevices to the power source for optimal PTE is also a challenging task as the microdevice orientation is not easily controlled during or post-injection (Khalifa et al., 2021a). These obstacles are exacerbated in wireless microdevices as the larger conventional IMDs are more tolerant to misalignments, both rotationally and directionally. Rotational-misaligned microdevices (those powered by RF or US in particular) will diminish their PTE, hence limiting their injection depths. Thankfully, novel receivers can alleviate these issues. For instance, sub-mm scale magnetoelectric (ME) antennas can be a great alternative to conventional wireless powering techniques (Zaeimbashi et al. 2021; Khalifa et al., 2021b, c; Alrashdan et al. 2021) -- such acoustically actuated ME antennas provide an ideal balance between miniaturization and PTE. Moreover, they are less sensitive to Tx-Rx misalignment and can potentially eliminate the need for matching networks. It should be noted that the optical I/O based microdevices are also less prone to misalignments as the tissue scattering provides a much gradual intensity gradient.

One of the main applications of injectable microdevices is to enable a distributed sensing/actuating network. Therefore, the ability to individually address the microdevices for communication and control is crucial. As the technology is still in its infancy, this challenge has yet to be addressed. Fortunately, a communication scheme that is scalable to large numbers of epicortical miniaturized implants has recently been demonstrated (Lee et al., 2021b). The authors claim that their proposed link configuration could potentially be scaled to 770 implants using a customized time-division multiple access protocol.

### Electrode integration

Another challenge in developing injectable microdevices as a neural interface is microelectrode integration. As stimulating microdevices become smaller, a reduction in the anode and cathode separation could make the stimulation less effective as it would require closer proximity to the target neurons for a given stimulus current. This problem can be mitigated by the microdevice aspect ratio where each electrode is placed on the microdevice-ends (as implemented in the MOTE). Another solution is to place the surface microelectrodes on the lateral surface (as implemented in the Microbead (Khalifa et al. 2017)). Another solution is to eliminate the need for electrodes by using different neural monitoring (e.g., calcium imaging (Zhang et al. 2020), magnetic sensors (Zaeimbashi et al. 2021)) and manipulation (e.g., optogenetics (Charthad et al. 2018; Montgomery et al. 2015) and magnetic (Khalifa, Zaeimbashi, et al. 2021d) techniques, but most are not (yet) applicable to clinical applications and do not offer the same spatial and temporal resolution as electrodes.

### Encapsulation

Packaging microdevices is a new challenge that needs to be seriously addressed for chronically injected microdevices. Robust encapsulation is required to prevent damages to the CMOS chip and to protect the tissue from the toxic species (e.g., Cu) in the CMOS chip. Conventional established techniques either significantly increase the total volume of the microdevice or hampers their wireless link. Fortunately, new packaging solutions that are biocompatible and offer ultra-thin encapsulation have recently been demonstrated such as: silicon carbide by PECVD deposition (Diaz-Botia et al. 2017), thermally grown silicon dioxide (Fang et al. 2016), and multi-layered ALD coating (Jeong et al. 2019).

### Post-injection migration

Although injectable microdevices are minimally invasive, regardless of the implantation techniques used, trauma to the brain tissue is unavoidable and the immune response will be activated during the injection. This also raises the issue that the implants might migrate through neural tissues over time, which led to a recent study in rat models (Khalifa et al., 2021a). Microdevices injected in different areas of the brain were tracked from 1 week to 4.5 weeks post-injection using a 9.4 T magnetic resonance imaging (MRI) scanner. The MR images showed that microdevices smaller than 0.01 mm^3^ remain stationary at the injection site in the brain. Based on microglia and astrocytes immunoreactivity to microdevices, the authors hypothesized that the glial scar formation around the microdevices prevented the migration of chronically injected microdevices in the brain. The findings are promising, however, it is yet to be investigated if microdevices migrate within the first week post-injection when the FBR is at its peak.

## Conclusion and future directions

Aggressive miniaturization of injectable microdevices provides new opportunities with distinct advantages in safety, longevity, and spatial resolution. Microdevices can also be extended to wireless sensing of other types of physiological parameters such as pH, oxygen, bicarbonate, and neurotransmitters. Once scaled down to a cellular level, microdevices can not only monitor metabolism in the bloodstream but also concurrently capture multiple physiological parameters in various areas in the human body (PNS and CNS) to create a distributed sensing/actuating network. These multidimensional physiological signals can constitute big data on human physiology to make truly personalized healthcare (e.g., prognosis and real-time therapeutic evaluation) possible and to enable long-term human model studies (e.g., metabolic syndrome and aging) that current clinical trials cannot provide.

While the latest injectable wireless microdevices show great promise in developing a research platform for animal models, significant technological challenges must be addressed before they could become applicable for clinical applications. Future efforts should focus on characterizing, in terms of efficacy, efficiency, and safety, the injection techniques presented in this review. There is also a lack of safety evaluations and chronic studies on injectable microdevices. Addressing these would bring the technology one step closer to clinical trials, and 1 day such microdevices would be able to change our healthcare landscape by revolutionizing therapeutics and diagnostics frameworks in humans.

## Data Availability

Not applicable.

## Abbreviations

IMDs: Implantable medical devices
CMOS: Metal-oxide-semiconductor
FBR: Foreign body response
PEG: Polyethylene glycol
ALD: Atomic layer deposition
PECVD: Plasma-enhanced chemical vapor deposition
CNS: Central nervous system
PNS: Peripheral nervous system
PPM: Pulse position modulation
ASIC: Application-specific integrated circuit
PTE: Power transfer efficiency
ME: Magnetoelectric
